# Factors Associated With Female Adolescent Usage of Modern Contraceptives in Northern Ghana: A Cross‐Sectional Survey of a Rural Municipality

**DOI:** 10.1002/puh2.70307

**Published:** 2026-06-29

**Authors:** Joseph Lasong, Yula Salifu, Gordon Dandeebo

**Affiliations:** ^1^ Department of Population and Reproductive Health, School of Public Health University for Development Studies Tamale Ghana; ^2^ Department of Sociology and Social Work, Faculty of Social Science and Arts SD Dombo University of Business and Integrated Development Studies Wa Ghana

**Keywords:** adolescent, female, Ghana, modern contraceptive use, rural

## Abstract

**Background:**

Modern contraceptive use is an effective strategy for reducing unintended adolescent pregnancies, sexually transmitted infections and unsafe abortions. Adolescent girls in rural populations are disproportionately affected by the adverse outcomes of contraceptive nonuse. This study examined factors associated with modern contraceptive use among female adolescents (FAs) in the Savelugu Municipality of Northern Ghana.

**Methods:**

A community‐based cross‐sectional study was conducted from June to August 2021 using systematic sampling to select 408 sexually active adolescent girls aged 15–19 years. Binary logistic regression was performed using SPSS version 25.0 to identify factors associated with modern contraceptive use. All statistical tests were two‐tailed, with *p* < 0.05 considered statistically significant.

**Results:**

The prevalence of modern contraceptive use among FAs was 20.8%. The majority (75.2%) had heard of modern contraceptives. Maternal formal education was associated with lower odds of contraceptive use (adjusted odds ratio [AOR] = 0.178, 95% confidence interval [CI]: 0.040–0.791). Source of contraceptive information (health worker/teacher vs. radio/peers/parents) was also associated with lower odds of use (AOR = 0.151, 95% CI: 0.036–0.640). These associations should not be interpreted as causal; they may reflect unmeasured confounding or reverse causality.

**Conclusions:**

Maternal education and source of contraceptive information were associated with adolescent contraceptive use in this rural setting. The unexpected negative associations may be explained by socially conservative contexts or unmeasured factors such as adolescent motivation and self‐efficacy. Given the wide CIs, these findings should be interpreted as preliminary and require confirmation in larger, adequately powered studies. Interventions should address social norms, strengthen mother–daughter communication and leverage mass media for destigmatising information.

## Graphical abstract

Among 408 sexually active adolescent girls in rural northern Ghana, modern contraceptive use was low (20.8%) despite high awareness (75.2%). Maternal education and information from health workers/teachers were associated with lower use, whereas clinic availability increased use. Interventions must move beyond awareness to address social norms and youth‐friendly service delivery.

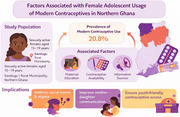

**Abbreviations**
AOR: adjusted odds ratio
CHPS: community‐based health planning and services
CI: confidence interval
FA: female adolescent
FP: family planning
GDHS: Ghana Demographic and Health Survey
GHS: Ghana Health Service
GSS: Ghana Statistical Service
SD: standard deviation
SRHR: sexual and reproductive health and rights
WHO: World Health Organization
YFS: youth‐friendly services

## 1 Introduction

Unplanned pregnancies among adolescent girls remain a significant global public health issue [1]. In developing nations, nearly 15 million female adolescents (FAs) aged 15–19 years give birth annually [2]. Sub‐Saharan African countries record the highest rates of adolescent pregnancy, with approximately one third ending in unsafe abortion [3]. In Ghana, younger women report more unintended pregnancies than older women [4]. Recent national data indicate that about 15% of FAs aged 15–19 years have ever been pregnant, 11% have given birth, and 4% have experienced pregnancy loss. Teenage pregnancy rates vary substantially by region, ranging from 6% in Greater Accra to 26% in the Savannah zones of Northern Ghana [5].

Low modern contraceptive use contributes to elevated rates of maternal mortality, teenage pregnancy, sexually transmitted infections and unsafe abortion [6]. In low‐ and middle‐income countries, family planning (FP) method use among teenagers is 31.6% [7]. The 2017 Ghana Maternal Health Survey reported 35.6% modern contraceptive use among sexually active FAs [8]. However, estimates from consecutive Demographic and Health Surveys show considerable variation: 44.4% in 2022, 43.7% in 2014 and 66.6% in 2008 [5, 9, 10].

Because most adolescents initiate sexual activity before age 18 years, strengthening adolescent reproductive health services is a priority [11]. Nevertheless, adolescents face substantial barriers to accessing FP services, including negative healthcare provider attitudes, privacy concerns, stigma and confidentiality challenges [12, 13, 14]. Factors influencing adolescent contraceptive use include education, employment, parental influence, parity, age, place of residence, marital status, religion and income [15, 16, 17].

Although Ghana's costed implementation plan has advanced FP services, limited access coupled with insufficient medical personnel continues to constrain contraceptive use [9, 16, 17, 18, 19, 20, 21, 22]. Most existing studies focus on contraceptive use among all reproductive‐aged women without detailed age‐stratified analysis [18, 19, 23] or rely on demographic and health survey data [6, 20, 21] and maternal health surveys [22]. Community‐based studies examining factors associated with adolescent contraceptive use, particularly in rural Northern Ghana, remain scarce. This gap is especially concerning given reports of low FP uptake among reproductive‐aged women in this region, especially those aged 15–19 years [5, 16, 17]. Accordingly, this study examined factors associated with modern contraceptive use among FAs in the Savelugu Municipality of Northern Ghana.

## 2 Materials and Methods

### 2.1 Study Population, Design and Area

The study focused on FAs aged 15–19 years, the age range during which most adolescents become sexually active and experience consequences such as unintended pregnancy [5]. Eligible participants were sexually active FAs aged 15–19 years (married or unmarried and in school or out of school), who had resided in the municipality for 6 months or more, agreed to participate and, if under 18 years, had parental or guardian consent. Exclusion criteria included nonagreement to participate, absence during data collection and, for those under 18 years, lack of parental or guardian consent.

The study employed a community‐based cross‐sectional design conducted from June to August 2021 in the Savelugu Municipality of Northern Ghana. Ghana is a West African country with a population exceeding 32 million and ranks among the top 10 African economies by GDP per capita (over $4500), though with considerable geographic variation in wealth. The Northern Region is one of Ghana's poorest regions, predominantly agricultural, and comprises 28 districts and municipalities. Savelugu Municipality is one of 26 administrative districts in the Northern Region [9]. According to the 2010 population and housing census, Savelugu has a population of 139,283, representing 5.1% of the region's total population. Most residents live in rural areas [9]. The municipality has five sub‐municipalities and nine health facilities, including a municipal hospital, three health centres, two clinics and three community‐based health planning and services (CHPS) compounds [24].

### 2.2 Sample Size and Sampling Technique

Sample size was calculated using Cochran's formula [25]: *N* ≥ *Z* ^2^ *p*(1*p*)/*d* ^2^, with a 95% confidence interval (CI) (*Z* = 1.96), an adolescent modern contraceptive use prevalence of 14.5% [20] and a marginal error of 0.05. The minimum sample size was 191. After allowing for a 10% nonresponse rate [6, 15, 20], the sample was increased to 210. To account for the clustering effect of the multistage sampling design (systematic sampling within communities), a design effect of 1.9 was applied, yielding a target sample of 399. Ultimately, 408 participants were recruited to increase statistical power. All recruited participants responded fully (response rate: 100%). The 100% response rate was because of the household substitution versus true response rate.

A systematic sampling technique was employed. First, simple random sampling was used to select one community from each of the five sub‐municipalities. Sample size was allocated proportionately to the estimated number of reproductive‐aged women in each community. Households in each community were labelled, and a sampling interval of four was used to select the first household. Every fourth household was consecutively selected until the sample size was reached. Within each selected household, an adolescent who met the inclusion criteria was sampled. Interviews were conducted in a secure environment to safeguard privacy. When a household contained more than one eligible adolescent, simple random selection was used to choose one respondent. When no eligible adolescent was found in a selected household, the next household was considered.

### 2.3 Data Collection Procedures

Five enumerators holding Master of Public Health degrees and fluent in English and various local dialects were trained for 2 days on data collection procedures. The structured questionnaire was adapted from previous literature [3, 6, 20, 22] and expert reviews, covering contraceptive use, socio‐demographic characteristics, familiarity with modern contraceptives and reproductive history.

#### 2.3.1 Dependent Variable

Ever used modern contraceptives (yes/no): Modern contraceptive use was defined as ever having used any of the following methods: pills, female sterilisation, intrauterine devices, injectables, implants, female condoms, diaphragms, emergency contraception or lactational amenorrhoea [9].

#### 2.3.2 Familiarity and Attitudes Assessment

Five questions assessed familiarity with and attitudes toward modern contraceptives: (1) Have you ever heard of modern contraceptives? (2) Is it true that first‐time pregnancy can occur after first sex? (3) Does modern contraceptive use during sex provide total protection from pregnancy? (4) Is modern contraceptive use a woman's decision? (5) Does modern contraceptive use cause promiscuity? Responses to each of the five questions were analysed descriptively. No composite score was calculated, as the items assess distinct constructs (exposure, factual knowledge and attitudes), and the measure was not validated as a scale. Importantly, this measure captures passive familiarity (having ‘heard of’) rather than comprehensive knowledge of method types, correct usage, side effects or access points.

The questionnaire was prepared in English and Dagbani. It was pretested among 40 FAs in a nearby community with similar characteristics to the study population. Before the main interviews, questionnaires were evaluated for completeness and comprehensiveness, translated from English to local dialects and backtranslated to ensure accuracy. This enabled non‐English‐speaking adolescents to understand and respond appropriately. Each interview lasted 20–45 min. The STROBE guidelines for cross‐sectional studies were followed for reporting.

### 2.4 Statistical Analysis

Data were cleaned and analysed using IBM SPSS version 25.0. Descriptive statistics were presented as frequencies, percentages, means and standard deviations (SDs). Bivariate analysis used chi‐square or Fisher's exact test. Variables with *p* < 0.1 in bivariate analysis were considered for inclusion in the multivariable logistic regression model. Multicollinearity was assessed using variance inflation factors (VIFs). Variables with VIF > 10 were excluded from the adjusted model. Specifically, ‘Ever participated in any family planning education or seminar’ had VIF > 10, indicating high collinearity with ‘source of information on modern contraceptives’ (as seminar participation is a specific information source); this variable was, therefore, excluded. Model fit was assessed using the Hosmer–Lemeshow goodness‐of‐fit test (*p* > 0.05 indicates acceptable fit). Backward stepwise elimination (likelihood ratio criterion) was applied, retaining variables that remained statistically significant (*p* < 0.05) or were considered a priori confounders on the basis of the literature. The final model fit was acceptable (*p* = 0.432). All statistical tests were two‐tailed, with *p* < 0.05 considered statistically significant.

## 3 Results

### 3.1 Socio‐Demographic and Reproductive Characteristics

Table 1 presents the frequency distribution and bivariate analysis of socio‐demographic variables by modern contraceptive use. The mean age of respondents was 17.59 years (SD = 1.287). Over half (58.3%) were aged >17 years. Mean age at first sexual intercourse was 14.79 years (SD = 1.69). More than half (52.9%) reported having a partner, and 83.8% had no more than one partner.

**TABLE 1:** Socio‐demographic and reproductive characteristics by modern contraceptive use.

		Modern contraceptive use		
Variable	Total *n* (%)	No *n* (%)	Yes *n* (%)	*p* value
**Age (mean ± SD: 17.59 ± 1.287)**				0.621
≤17 years	170 (41.7)	137 (80.6)	33 (19.4)	
>17 years	238 (58.3)	186 (78.2)	52 (21.8)	
**Educational status**				^a^
Formal education	400 (98.0)	321 (80.3)	79 (19.8)	
No formal education	8 (2.0)	2 (25.0)	6 (75.0)	
**Religion**				1.000
Christian/Traditionalist	168 (41.2)	133 (79.2)	35 (20.8)	
Islam	240 (58.8)	190 (79.2)	50 (20.8)	
**Marital status**				0.557
Married	41 (10.0)	34 (82.9)	7 (17.1)	
Not married	367 (90.0)	289 (78.7)	78 (21.3)	
**Living arrangement**				0.034
Parents/Guardian	363 (89.0)	293 (80.7)	70 (19.3)	
Partner/Self	45 (11.0)	30 (66.7)	15 (33.3)	
**Father's education**				0.222
Formal education	182 (44.6)	139 (76.4)	43 (23.6)	
No formal education	226 (55.4)	184 (81.4)	42 (18.6)	
**Mother's education**				0.024
Formal education	122 (29.9)	88 (72.1)	34 (27.9)	
No formal education	286 (70.1)	235 (82.2)	51 (17.8)	
**Father's employment**				1.000
Employed	404 (99.0)	320 (79.2)	84 (20.8)	
Unemployed	4 (1.0)	3 (75.0)	1 (25.0)	
**Mother's employment**				0.024
Employed	306 (75.0)	234 (76.5)	72 (23.5)	
Unemployed	102 (25.0)	89 (87.3)	13 (12.7)	
**Ever used modern contraceptives**				
No	323 (79.2)	—	—	
Yes	85 (20.8)	—	—	
**Age at first sex (mean ± SD: 14.79 ± 1.69)**				0.007
≤15 years	291 (71.3)	233 (80.1)	58 (19.9)	
>15 years	117 (28.7)	75 (64.0)	42 (36.0)	
**Has a partner**				0.020
No	192 (47.1)	162 (84.4)	30 (15.6)	
Yes	216 (52.9)	161 (74.5)	55 (25.5)	
**Number of partners**				0.674
≤1	181 (83.8)	136 (75.1)	45 (24.9)	
>1	35 (16.2)	25 (71.4)	10 (28.6)	
**Partner's education**				0.032
Formal education	170 (78.7)	129 (75.9)	41 (24.1)	
No formal education	46 (21.3)	32 (69.6)	14 (30.4)	
**Ever attended FP education/seminar**				<0.001
No	143 (35.0)	132 (92.3)	11 (7.7)	
Yes	265 (65.0)	191 (72.1)	74 (27.9)	
**Sex education with friends/family**				0.442
No	329 (80.6)	263 (79.9)	66 (20.1)	
Yes	79 (19.4)	60 (75.9)	19 (24.1)	
**Ever had an abortion**				0.005
No	340 (83.3)	278 (81.8)	62 (18.2)	
Yes	68 (16.7)	45 (66.2)	23 (33.8)	

*Note:* The apparent significant association for educational status is an artefact of the very small number of respondents with no formal education (*n* = 8); this variable was not retained for multivariate analysis.

Abbreviations: FP, family planning; SD, standard deviation.

^a^Fisher's exact test used.

In bivariate analysis, maternal education (*p* = 0.024), mother's employment status (*p* = 0.024), history of abortion (*p* = 0.005), living arrangement (*p* = 0.034), having a partner (*p* = 0.020), ever attended FP education (*p* < 0.001), partner's educational status (*p* = 0.032) and age at first sex (*p* = 0.007) were significantly associated with modern contraceptive use.

### 3.2 Familiarity With Modern Contraceptives

Nearly one‐quarter (24.8%) had never heard of contraceptives. Among all respondents, 75.2% had heard of modern contraceptives, though 43.6% believed that contraceptive use causes promiscuity. Among those who had heard (75.2%), 58.0% obtained information from health workers or teachers. Source of contraceptive information was significantly associated with modern contraceptive use (*p* < 0.001) (Table 2). Among those who use contraceptives 35.3% received it from clinic/health facility (Table 3).

**TABLE 2:** Familiarity with modern contraceptives by use status.

		Modern contraceptive use		
Variable	Total *n* (%)	No *n* (%)	Yes *n* (%)	*p* value
**Ever heard of contraceptives**				<0.001
No	101 (24.8)	101 (100.0)	0 (0.0)	
Yes	307 (75.2)	222 (72.3)	85 (27.7)	
**Source of information**				<0.001
Health worker/Teacher	180 (58.6)	139 (77.2)	41 (22.8)	
Radio/Peers/Parents	127 (41.4)	83 (65.4)	44 (34.6)	

*Note:* An adolescent may be aware that contraceptives exist (passive familiarity) but lack the knowledge, skills and support to obtain and use them correctly. This distinction is critical for explaining the low uptake.

**TABLE 3:** Source of contraceptives among users (*n* = 85)—Descriptive finding only.

Source	Frequency (*n*)	Percentage
Clinic/Health facility	30	35.3
Drug store/Pharmacy	55	64.7
**Total**	**85**	**100.0**

### 3.3 Factors Associated With Modern Contraceptive Use

Multivariate analysis (Table 4) revealed two significant factors:

1. **Maternal education**: FAs whose mothers had formal education were approximately 82% less likely to use modern contraceptives compared to those whose mothers had no formal education (adjusted odds ratio [AOR] = 0.178, 95% CI: 0.040–0.791, *p* = 0.023).
2. **Source of contraceptive information**: Adolescents who heard about contraceptives from health workers or teachers were approximately 85% less likely to use modern contraceptives compared to those who heard from radio, peers or parents (AOR = 0.151, 95% CI: 0.036–0.640, *p* = 0.010).

**TABLE 4:** Multivariate analysis of factors associated with modern contraceptive use.

Variable	AOR	95% CI	*p* value
**Mother's education**			
Formal education	0.178	0.040–0.791	0.023
No formal education	Reference		
**Source of information**			
Health worker/Teacher	0.151	0.036–0.640	0.010
Radio/Peers/Parents	Reference		
Mother's employment (employed vs. unemployed [reference])	1.621	0.335–7.846	0.548
Ever had an abortion (No vs. Yes [reference])	1.125	0.232–5.460	0.884
Age at first sex (≤15 vs. >15 years [reference])	0.139	0.019–1.002	0.050
Partner's education (formal vs. no formal [reference])	1.171	0.270–5.088	0.833
Respondent's education (formal vs. no formal [reference])	1.001	0.901–1.010	0.099

*Note:* Variables with VIF > 10 (having a partner, ever attended family planning education/seminar and living arrangement) were excluded from the adjusted model. The variable ‘ever heard of contraceptives’ was perfectly predicted non‐use (0% use among those who never heard) and was therefore excluded from the multivariable model to avoid estimation instability. The wide confidence interval for age at first sex (0.019–1.002) indicates some statistical instability, likely due to the relatively small number of contraceptive users (*n* = 85); this should be interpreted as nonsignificant.

Abbreviation: AOR, adjusted odds ratio.

## 4 Discussion

This study examined factors associated with modern contraceptive use among FAs in rural Northern Ghana. A substantial proportion of participants (75.2%) reported having heard of modern contraceptives, whereas only 20.8% reported use. This difference between passive awareness and actual use is particularly pronounced in rural northern Ghana, where social norms, stigma and health system barriers may constrain translation of awareness into behaviour. Two factors were statistically associated with contraceptive use: maternal education (negatively associated) and source of contraceptive information (with formal sources paradoxically associated with lower use).

The association between age at first sexual intercourse and contraceptive use (AOR = 0.139; 95% CI: 0.019–1.002; *p* = 0.050) is borderline and not statistically significant at the conventional 0.05 threshold, as the CI includes 1.00. Therefore, this finding should be interpreted as suggestive rather than conclusive evidence of an association.

### 4.1 Maternal Education

The observed negative association between maternal formal education and adolescent contraceptive use (AOR = 0.178) was unexpected, as prior studies have generally found maternal education to be positively associated with adolescent reproductive health outcomes. However, in socially conservative rural contexts such as Northern Ghana, educated mothers may paradoxically impose stricter controls on adolescent daughters’ perceived sexual behaviour. Higher maternal education may be accompanied by heightened concerns about family reputation, moral standing and adherence to traditional norms regarding premarital sexual activity. Consequently, educated mothers might actively discourage contraceptive use due to the belief that contraceptive availability would ‘enable’ sexual activity. Alternatively, educated mothers may communicate more effectively with daughters about delaying sexual debut rather than about contraceptive methods. This study did not collect data on parent–child communication, household decision‐making dynamics or maternal attitudes toward adolescent sexuality; therefore, the mechanisms underlying this association cannot be determined from the present data. The finding requires replication in other rural samples and further investigation using mixed methods to explore potential contextual moderators, such as community norms around adolescent sexuality or intergenerational communication patterns specific to northern Ghana.

### 4.2 Source of Information

The finding that adolescents who reported health workers or teachers as their source of contraceptive information had lower odds of use (AOR = 0.151) should be interpreted cautiously. This association may reflect unmeasured confounding by adolescent motivation, agency or self‐efficacy, rather than a deficiency in formal information sources per se. It is possible that adolescents who are more motivated or self‐efficacious to use contraceptives may seek information from multiple sources, including peers and media, whereas those who rely solely on formal channels may face additional barriers. Alternatively, reverse causality may be at play: Adolescents who are already using contraceptives may have less need to seek information from health workers or teachers. This study did not assess the quality, tone or context of these interactions, nor did it measure adolescents’ perceptions of provider attitudes or service accessibility. As such, attributing this association to factors, such as judgemental attitudes or lack of youth‐friendly services (YFS), cannot be confirmed by the present data. Further research incorporating measures of service quality, confidentiality and adolescent–provider interactions is needed to clarify these pathways.

### 4.3 Nonsignificant Findings

Marital status, history of abortion and several other variables were not significantly associated with contraceptive use in the multivariate model. These findings align with some studies [26, 27, 28, 29] but differ from others [30, 31, 32, 33, 34]. Differences may reflect contextual factors, such as strong social expectations for married couples to have children soon after marriage, limited autonomy of married adolescent girls in reproductive decision‐making or the possibility that adolescents with abortion history may view termination as a primary method for managing unintended pregnancies [35].

### 4.4 Strengths and Limitations

This study contributes to the limited literature on FA contraceptive use in rural northern Ghana, providing evidence to inform policy interventions for marginalised communities. However, several limitations should be acknowledged. First, because contraceptive use is stigmatised among adolescents and was self‐reported, underreporting, response bias and social desirability bias are possible; high confidentiality was maintained to mitigate this. Second, the cross‐sectional design precludes causal inference. Third, the single municipality sample limits generalisability to the entire region or country. Fourth, the wide CIs observed for some variables (e.g., age at first sex: 95% CI 0.019–1.002) indicate statistical instability, likely due to the relatively small number of contraceptive users (*n* = 85). Fifth, our familiarity measure captured basic passive familiarity rather than comprehensive knowledge of method types, correct usage, side effects or access points; a more nuanced measure might yield different results. Sixth, the study did not include other potentially relevant parameters such as perceptions of contraceptive use or risky sexual behaviours. Seventh, the study did not systematically record the number of potentially eligible adolescents under 18 years who were excluded because parental or guardian consent could not be obtained. This omission prevents quantitative assessment of selection bias. However, if excluded adolescents were more likely to be sexually active without parental knowledge, the study may have under‐represented the most hidden and potentially highest need subgroup. Eighth, the study may have been underpowered for some subgroup analyses, particularly given that the sample size estimation was based on a lower prevalence from a broader adolescent population rather than sexually active adolescents. This may partly explain the wide CIs observed. Mixed methods research is needed to validate and extend these findings.

## 5 Conclusions

This study revealed that although 75.2% of FAs in rural northern Ghana had heard of modern contraceptives, only 20.8% reported use. Maternal formal education (negatively associated) and source of contraceptive information (health worker/teacher negatively associated relative to radio/peers/parents) were the key factors. These associations should not be interpreted as causal; they may reflect unmeasured confounding, reverse causality or contextual factors unique to this rural setting. Given the wide CIs surrounding the key effect sizes (reflecting limited precision due to the modest number of contraceptive users, *n* = 85), these findings should be interpreted as preliminary and require confirmation in larger, adequately powered studies that enrol exclusively sexually active adolescents. Moreover, this study is hypothesis‐generating, and the wide CIs may suggest that the effect sizes are imprecise.

On the basis of these findings, we recommend:

1. **Implement community‐based interventions to facilitate mother–daughter sexual and reproductive health and rights (SRHR) communication**: The negative association between maternal education and adolescent contraceptive use suggests that maternal education alone may not be sufficient to influence adolescent contraceptive use. The Ministry of Gender, Children and Social Protection, in partnership with NGOs, should pilot community‐based programmes using trusted community health nurses or peer educators as facilitators to guide mother–daughter conversations on sexual and reproductive health, addressing social norms, building communication skills and destigmatising contraceptive use.
2. **Leverage mass media for destigmatising information**: Adolescents who heard about contraceptives from radio, peers or parents had higher use than those who heard from health workers or teachers. Mass media (radio and social media) should disseminate destigmatising, youth‐friendly contraceptive information that normalises contraceptive use among adolescents.
3. Qualitative research to understand this negative association between source of information and contraceptive use, as well as task‐shifting to peer educators to improve contraceptive uptake.

## Author Contributions

Yula Salifu and Joseph Lasong conceived and designed the study. Yula Salifu, Joseph Lasong and Gordon Dandeebo wrote the manuscript. Yula Salifu and Joseph Lasong edited and reviewed the manuscript. Yula Salifu, Joseph Lasong and Gordon Dandeebo performed statistical analyses and interpreted the results. All authors proofread the manuscript, contributed to discussion and approved the final version.

## Funding

The authors have nothing to report.

## Ethics Statement

Ethical approval was obtained from the University for Development Studies Ethics Review Board (UDS/RB/date: 07/05/2021). Permission was also obtained from the Northern Regional Health Directorate and Savelugu Municipal Health Administration. The principles of the Helsinki Declaration were followed.

## Consent

Informed consent was obtained from all respondents. For adolescents under 18 years, parental or guardian consent was obtained, and the adolescent's assent was secured. Respondents aged 18 years and above gave consent directly. Participants were informed that participation was voluntary and that they could refuse to answer any question. Strict anonymity and confidentiality were assured.

## Conflicts of Interest

The authors declare no conflicts of interest.

## Data Availability

The data that support the findings of this study are available from the corresponding author upon reasonable request.
